# A novel isothermal whole genome sequencing approach for Monkeypox Virus

**DOI:** 10.1038/s41598-024-73613-3

**Published:** 2024-09-27

**Authors:** Matthias Licheri, Manon Flore Licheri, Lukas Probst, Cora Sägesser, Pascal Bittel, Franziska Suter-Riniker, Ronald Dijkman

**Affiliations:** 1 Institute for Infectious Diseases, University of Bern, Bern, Switzerland; 2 Graduate School for Cellular and Biomedical Sciences, University of Bern, Bern, Switzerland; 3 Multidisciplinary Center for Infectious Diseases, University of Bern, Bern, Switzerland; 4 European Virus Bioinformatics Center (EVBC), Jena, Germany

**Keywords:** Monkeypox virus, Mpox, Whole-genome sequencing, MDA, DNA sequencing, Next-generation sequencing, Pox virus

## Abstract

Monkeypox virus (MPXV) is the zoonotic agent responsible for mpox, an often-self-limiting pox-like disease. Since May 2022, an outbreak characterized by increased human-to-human transmission was detected outside the endemic regions. Whole genome sequencing (WGS) has been successfully used to keep track of viral evolution during outbreaks or for surveillance of multiple pathogens of public health significance. Current WGS protocols for MPXV are either based on metagenomic sequencing or tiled-PCR amplification. The latter allows multiplexing due to the efficient enrichment of the viral DNA, however, mutations or the presence of different clades can negatively influence genome coverage yield. Here, we present the establishment of a novel isothermal WGS method for MPXV based on Phi29 DNA polymerase-based multiple displacement amplification (MDA) properties making use of only 6 primers. This approach yielded from 88% up to 100% genome coverage using either alkaline denatured extracted DNA or clinical material as starting material, with the highest coverage generated by clinical material. We demonstrate that this novel isothermal WGS protocol is suitable for monitoring viral evolution during MPXV outbreaks and surveillance in any conventional laboratory setting.

**Supplementary Information:**

The online version contains supplementary material available at 10.1038/s41598-024-73613-3.

## Introduction

Monkeypox virus (MPXV) is a large, enveloped double-stranded DNA virus belonging to the genera *Orthopoxvirus* of the *Poxviridae* family^1^. It causes mpox, a viral disease with high fever, headaches, lymphadenopathy, and skin lesions resembling the ones of smallpox. The disease has a case fatality rate between 3.6% and 10.6%, depending on the infecting viral clade^2,3^. MPXV was first described in captive macaques in Denmark in 1958, and later, it was only first described in humans in 1970 in the Democratic Republic of Congo^4,5^. From that point, multiple outbreaks have been recorded in countries located in western and central Africa^3^. Transmission happens mainly in rural areas and is likely due to contact with an animal reservoir, such as small rodents and squirrels, with rare direct human-to-human transmission^1,6^.

Except for a smaller outbreak due to contact with infected imported animals in the United States of America in 2003, MPXV infections were limited to Africa, where MPXV is divided into two clades: clade I and clade II, of which the latter can be further divided into clade IIa and IIb^1,7^. Strains of the clade IIb were found responsible for the outbreak in Nigeria in 2017–2018, which already had increased human-to-human transmission clusters when people were in close contact^6^. In May 2022, an MPXV clade IIb was detected in the United Kingdom, and until January 3, 2024, it has led to more than 92,000 infections and 171 deaths globally in over 116 countries (www.who.org). The MPXV strain responsible for this recent global outbreak (hMPXV1) is characterized by a higher human-to-human transmission rate, especially in Men who have sex with Men (MSM) population, and a higher mutation rate possibly caused by the human apolipoprotein B mRNA-editing catalytic polypeptide-like 3 (APOBEC3) enzymes^8–10^.

Viral genomic information is essential to keep track of viral evolution, allowing better response to outbreaks, geographical tracking, vaccine design, and the identification of mutations of interest, such as those affecting antiviral treatments or pathogenicity^1,9^. However, most whole genome sequencing (WGS) protocols are based on either a metagenomic approach, which can be cost-intensive, or a tiled-PCR approach^9,11–13^. The latter has been successfully used for WGS of Zika virus and more recently for Severe Acute Respiratory Syndrome Coronavirus 2 (SARS-CoV-2), where the primer pools composed of approximately 100 primers had to be changed over time as mutations in the binding regions were detected^14,15^. Current tiled-PCR protocols for MPXV WGS use 98 and 326 primers, respectively, which anneal to the genome starting from more than 300 base pairs of the genomic ends, leaving part of the genome untargeted^11,12^. This large number of primers makes these protocols more susceptible to mutations, as shown for SARS-CoV-2, as well as limited to the viral strain or clade the primers were designed for^16–21^.

**Fig. 1:**
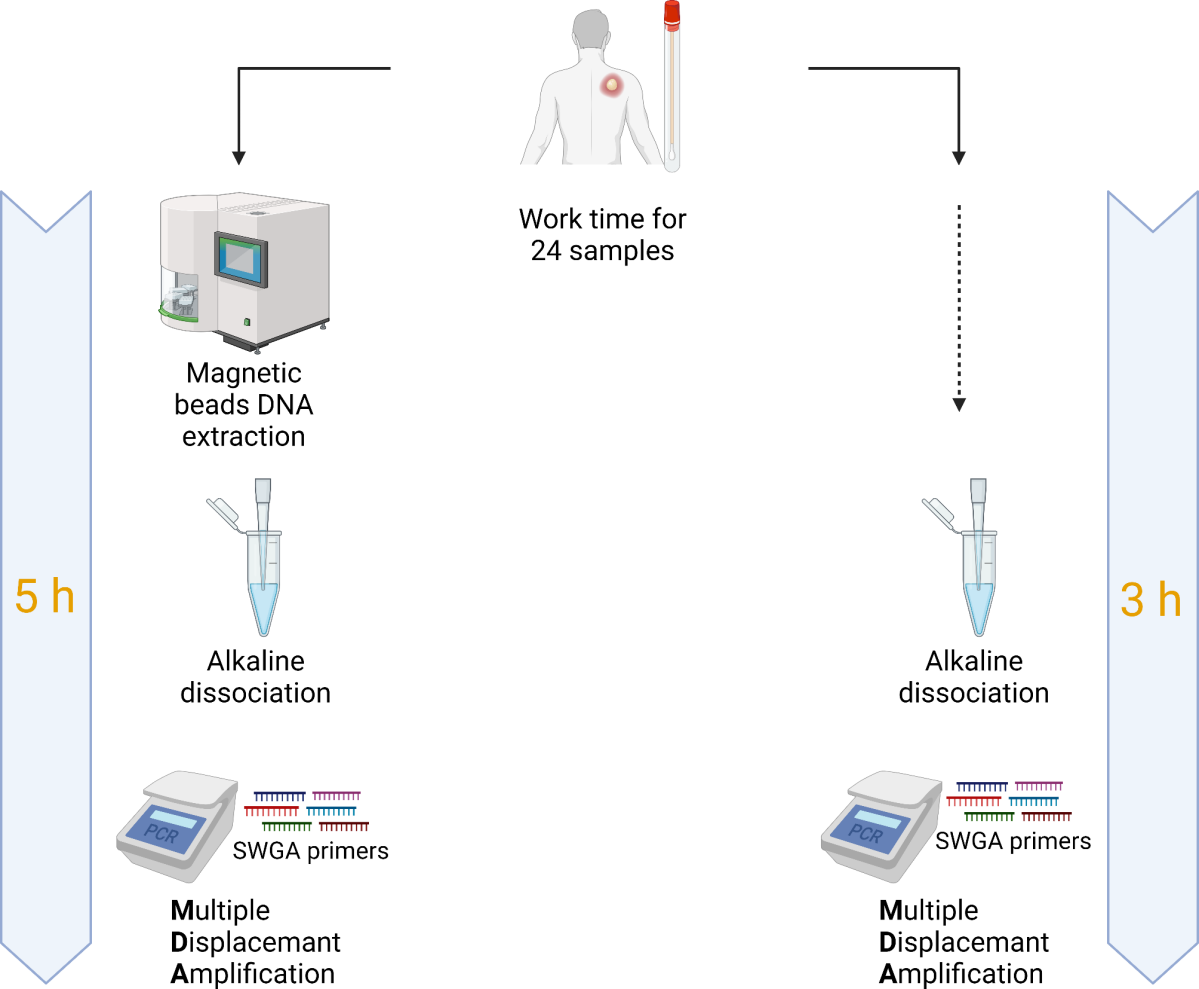
Schematic overview of the two amplification workflows used in this study. Comparison of the two protocol workflows using either direct clinical material or extracted DNA as input. On the left the samples were extracted using an automated HMW DNA extraction system, followed by alkaline lysis buffer incubation and amplification, whereas samples on the right were directly lysed and then amplified. For both the workflows the amount of time needed for 24 samples is shown. Created in BioRender. Licheri, M. (2024) BioRender.com/g83j532.

To overcome these limitations, we developed a novel isothermal WGS approach for MPXV based on the multiple displacement amplification (MDA) properties of the Phi29 DNA polymerase using only 6 primers (Fig. 1). We used the novel protocol to amplify extracted DNA and the clinical sample thereof that were previously denatured with an alkaline buffer as starting materials, yielding a mean coverage of 94%, with the clinical samples achieving the highest coverages. This demonstrates that this protocol can be used for surveillance and monitoring of viral evolution during MPXV outbreaks in any conventional laboratory setting.

## Results

**Fig. 2:**
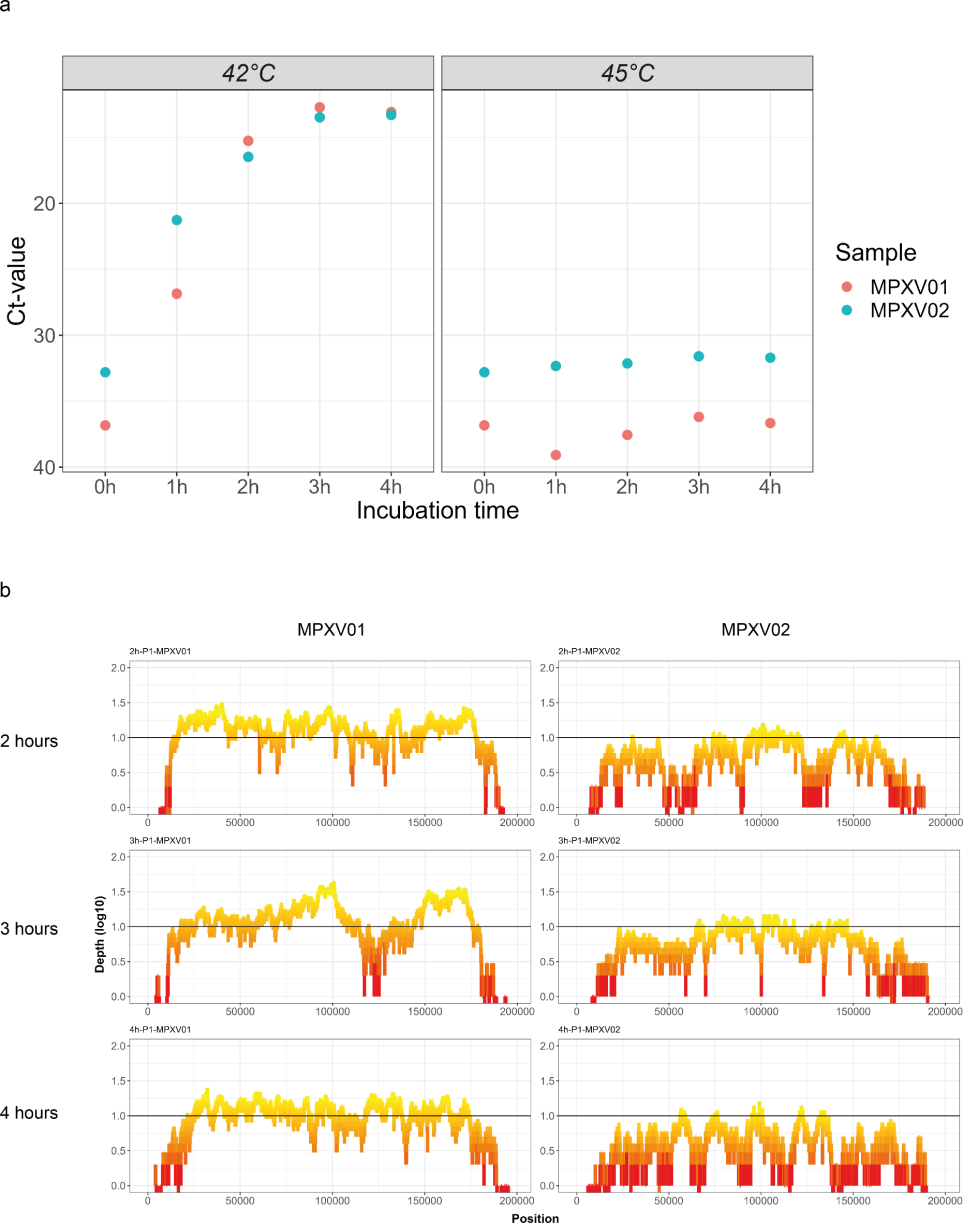
MPXV whole-genome amplification optimization. (a) To optimize the amplification reaction, DNA extracted from two different MPXV samples (red and blue) was incubated at either 42–45 °C for 1, 2, 3, or 4 h. The 0 h corresponds to the viral load at the start of the reaction. After amplification, all the samples were analyzed by qPCR, and the corresponding Ct-value was plotted (y-axis). (b) To compare the overall coverage of the samples amplified for 2, 3, and 4 h at 42 °C, the sequencing depth (log10 scale, y-axis) is plotted in function of each nucleotide position (x-axis) for both MPXV01 (left column) and MPXV02 (right column). The black horizontal line corresponds to 10 times sequencing coverage.

Because current tiled-PCR WGS protocols for MPXV are susceptible to mutations as well as relatively time-consuming and cost-intensive, we developed a novel method which uses the Phi29 DNA polymerase-based MDA. Using the swga2.0 command-line tool, we designed primers that preferentially target hMPXV1 over the human chromosomal and mitochondrial DNA. From the generated list of possible primer sets, we selected 6 octamers that in silico did not align with human mitochondrial DNA, as this would be more efficiently amplified compared to MPXV’s linear genome by the Phi29 polymerase. Each of the six octamers has more than 10 different annealing sites across the hMPXV1 genome (Supplementary Table S1). To assess the optimal reaction parameters for the recombinant Phi29 polymerase, we tested two incubation temperatures (42 and 45 °C) in combination with 4 incubation times (1, 2, 3, and 4 h) using the extracted DNA of 2 different hMPXV1 isolates (MPXV01 and MPXV02) followed by quantifying the yield by qPCR. This demonstrated that compared to the control (0 h), the genomic yield for samples incubated at 42 °C increased to a Ct-value of 15 after 2 h for both isolates and reached a plateau beyond this time point (Fig. 2a). In contrast, there was no increase in the genomic yield for samples incubated at 45 °C (Fig. 2a). Therefore, we only evaluated the sequence coverage for the samples incubated at 42 °C. This revealed that only a few reads were generated for hMPXV1 incubated for 1 h, resulting in an overall low coverage. Interestingly, samples incubated for 2, 3, or 4 h revealed similar results and generally reached genome coverages up to 90% (Fig. 2b). This reflects the reaction characteristics, which after a prolonged incubation time can lead to complete saturation of the reaction, resulting in no further or diminished amplification of the target. Based on these results, we therefore chose to use the MDA-based amplification protocol at 42 °C with only 2 h of incubation for all further optimization steps, as longer incubation did not yield significant differences.

To improve the sequencing coverage of the viral genome ends, we designed an octameric primer (MPXV_ter2) targeting the first and last 250 nucleotides (Supplementary Table S1) and evaluated different primer pools. Namely, primer pool 1 (P1) containing all 6 octamers designed with the swga2.0 tool, primer pool 2 (P2) where the MPXV_ter2 octamer is added, and an identical primer pool 3 (P3) where MPXV_05, which is predicted to form a dimer with itself in-silico, was omitted. To determine if adding the MPXV_ter2 increases the overall coverage of both genomic ends, we calculated the sequencing coverage and depth for the first and last 5 kb of the MPXV genome and compared this to the initial primer pool (P1) without the custom-designed octamer for the terminal ends. To accommodate for the difference in the number of sequencing reads for each sample, we first subsampled the reads before alignment and thereafter extracted the mean depth and coverage of the first and last 5 kb of the MPXV genome. This revealed that adding the custom-designed octamer increased the coverage of the first and last 5 kb of the MPXV genome (Table 1), while the overall sequencing coverage in the middle region of the MPXV genome was not negatively influenced by the addition of the MPXV_ter2 or the omission of MPXV_05. Based on these findings, we chose the P3 primer pool for further analysis, due to its slightly better performance compared to P2 in the recovery of the viral genomic ends.

**Table 1:** Assessment of genome termini amplification by the addition of a terminal primer. To improve the amplification of the genomic ends, the addition of a primer binding also at the genome termini was assessed by amplifying two MPXV samples using 3 different primer pools (P1, P2, P3). The first and last 5 kb of the genome were isolated from the consensus sequence and the number of nucleotides with at least 1x depth counted, showing a general coverage improvement of the two pools including the terminal primer (P2 and P3) compared to the amplification without (P1). The mean depth for the covered bases was also calculated.

Region		P1_MPXV01	P1_MPXV02	P2_MPXV01	P2_MPXV02	P3_MPXV01	P3_MPXV02
5’-end (5000 bp)	Coverage (> 0)	11.5	9.5	58.4	3.8	71.7	8.8
	Mean depth	0.1	0.1	0.6	0.0	3.3	0.1
3’-end (5000 bp)	Coverage (> 0)	0.0	0.0	31.8	97.2	98.2	21.9
	Mean depth	0.0	0.0	0.4	1.0	2.0	0.2

**Fig. 3:**
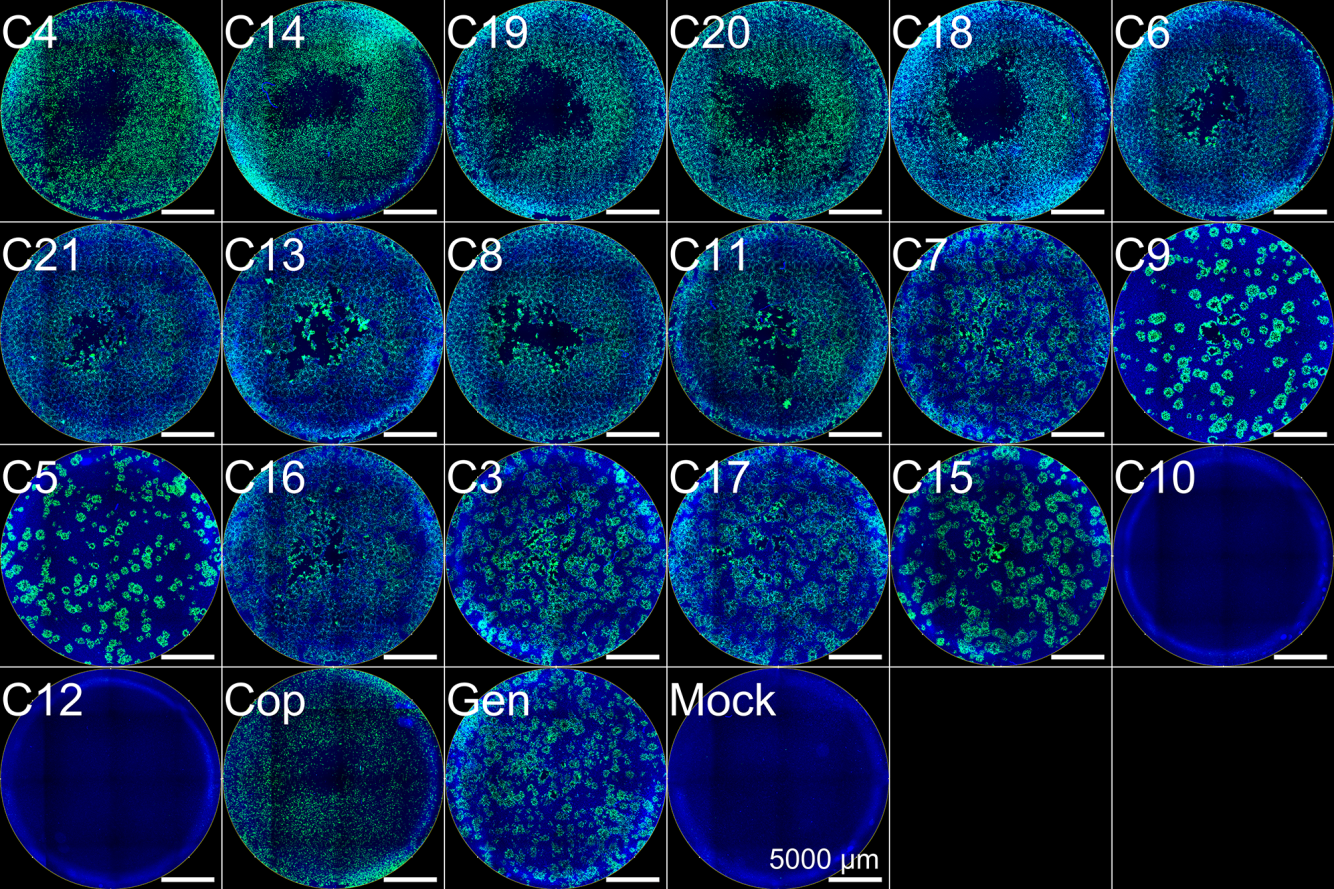
Immunofluorescence staining of MPXV-infected Vero cells. Vero cells were inoculated with the different clinical samples, two positive controls (Cop, Gen), and one negative control (mock, virus transport media). At 3 days post-infection, Vero cells were formalin-fixed and stained for immunofluorescence analysis to detect MPXV-infected cells using an antibody against the A27L protein of Vaccinia Virus  (anti-A27L, green) and DAPI to counterstain the cell nuclei (blue). The scale bar is 5000 μm. The images are arranged by corresponding Ct-value from the lowest (top left; Ct-value: 22.2) followed by each higher one on its right (highest Ct-value: 45).

Initial optimization was performed on extracted DNA from two clinical MPXV isolates, however, to further evaluate the method directly on clinical material, we used 19 clinical samples from which enough material was still available. Following DNA extraction, we performed a qPCR to determine the relative viral load for these clinical samples, which ranged from a Ct-value of 22.2 to 42 (Supplementary Table S2), whereas sample MPXV12 was negative (Ct-value ≥ 45). In addition, we also inoculated Vero cells with the clinical material to determine how this relates to infectious virus, as qPCR itself cannot discriminate between infectious and non-infectious viral particles. This revealed that MPXV could be isolated from 17 of the 19 samples (Fig. 3), which all had a Ct-value ≤ 31 (Supplementary Table S2). Interestingly, the degree of cytopathogenic effect (CPE) induced by MPXV is likely related to the Ct-value, as samples with a Ct-value below 27 resulted in full-blown CPE illustrated by a fluorescence signal across the well in the immunostaining. In contrast, samples with Ct-values between 27 and 31, showed individual plaques (Fig. 3). Combined, this illustrates that samples with a Ct-value ≤ 31 contain infectious viruses and are likely suitable for our MPXV WGS protocol.

**Fig. 4:**
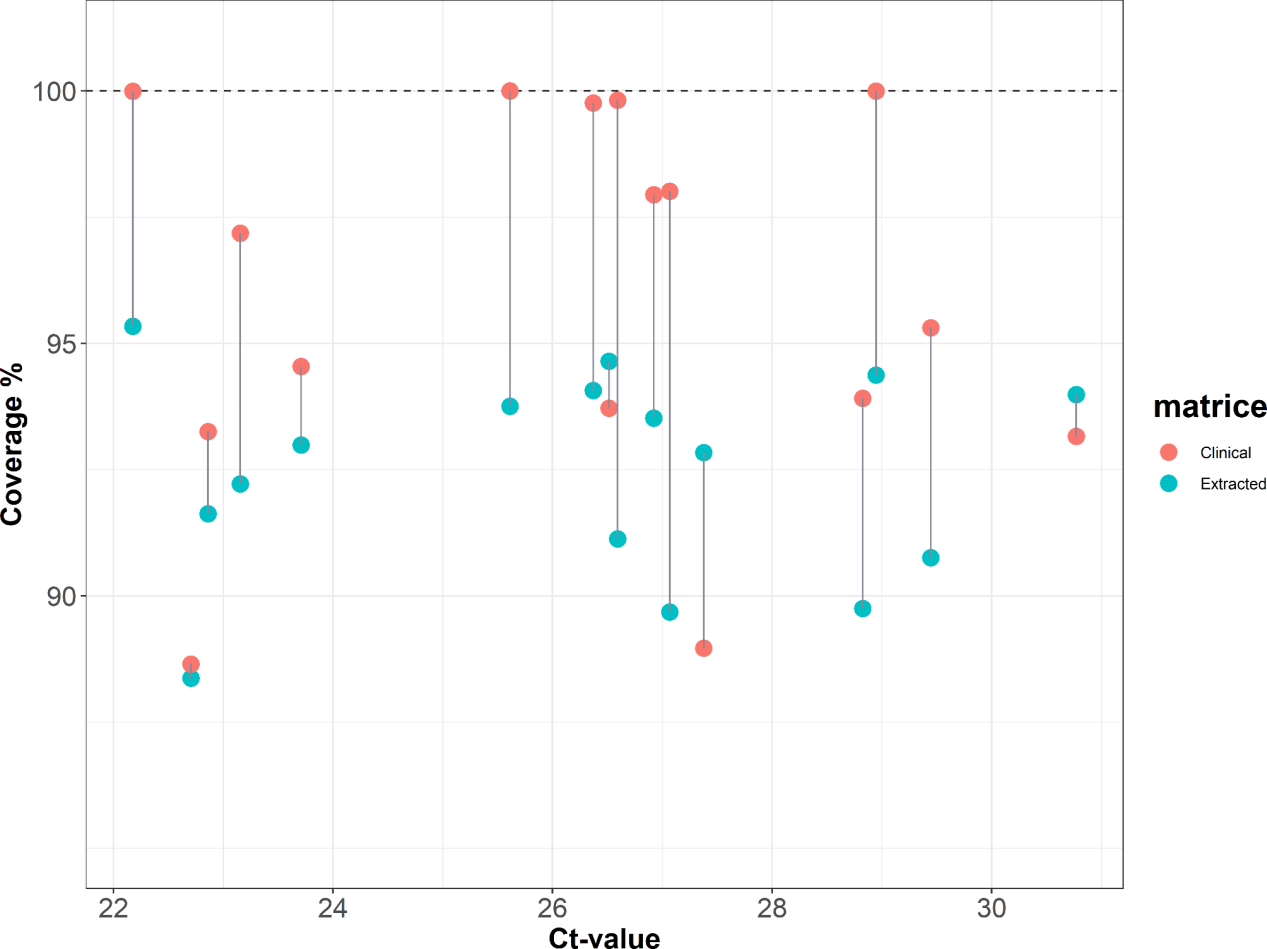
Comparison of the coverages achieved using either clinical sample or extracted DNA as input ordered by Ct-value. After sequencing, the percentages of coverage when using the clinical sample (red) and the extracted DNA (blue) as input are compared (y-axis, dashed line at 100% coverage). The vertical black line connects points of matrices belonging to the same sample of origin. The samples are ordered by their Ct-value (x-axis).

**Fig. 5:**
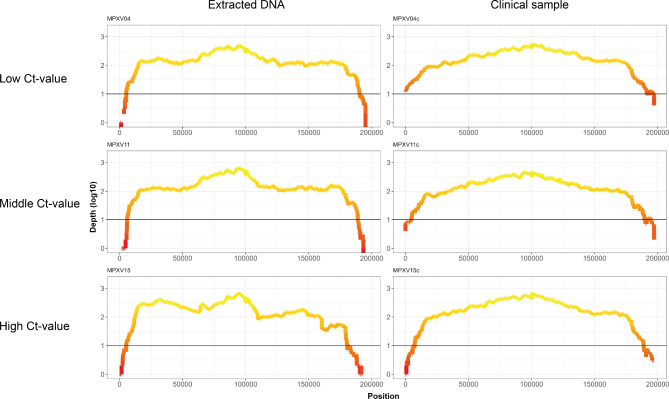
Coverage plots across MPXV genome comparing samples amplified from extracted DNA and clinical sample. To compare the overall coverage of each sample, the achieved sequencing depth (log10 scale, y-axis) is plotted in function of each nucleotide position (x-axis) for the samples with the lowest (22.2), mid (26.9), and highest (30.8) Ct-values using extracted DNA (left column) and clinical material (right column). The black horizontal line corresponds to 10 times coverage.

During the optimization of a Phi29-based WGS protocol for African Swine Fever (ASF) virus (Licheri et al., manuscript in preparation), we identified that an alkaline denaturing step with extracted DNA improves the overall percentage of viral reads, as well as the genome coverage. Therefore, we also incorporated this step in our current Phi29-based WGS protocol for MPXV. However, because an alkaline lysis was already used to release DNA from bacterial cells, we evaluated whether we could omit the nucleic acid purification step by directly using clinical material as input for the Phi29-based WGS protocol^22^. To this extent, we used the extracted DNA or the native samples from all 19 clinical samples as input for the Phi29-based MDA reaction for subsequent WGS. Irrespective of the input material, this led to the recovery of a genome coverage ranging from 88.4 to 100% for 16 of the 19 MPXV-positive samples. No consensus sequences were generated for MPXV10 and MPXV12 samples, likely due to the low viral load and absence of infectious virus (Supplementary Table S2, Fig. 3). Interestingly, despite that MPXV could be isolated from sample MPXV17, the genomic coverage was only around 36%. This likely is influenced by the overall low percentage of viral reads compared to the other samples (Supplementary Table S2). Nonetheless, when we compared the genomic coverage from the two different matrices, we observed that 13 out of the 16 samples had a better overall coverage when using clinical material as input for the Phi29-based WGS protocol (Fig. 4, Supplementary Table S2), regardless of the relative viral load (Fig. 5, Supplementary Fig. S1, Supplementary Table S2). In conclusion, this indicates that our methodology can resolve the whole-genome of MPXV using extracted nucleic acids or clinical material as input.

**Fig. 6:**
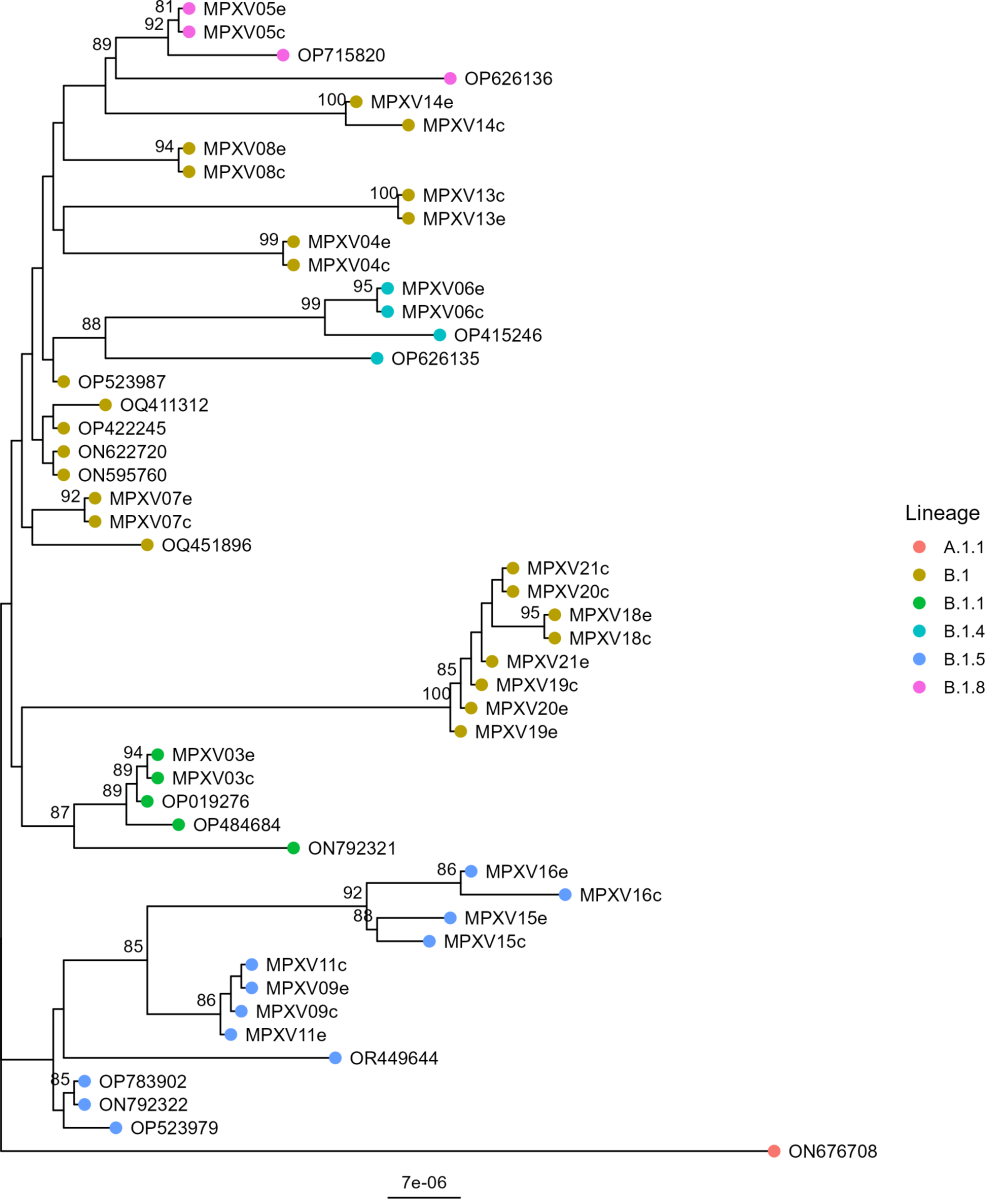
Phylogenetic tree of MPXV whole-genome sequences. Maximum-likelihood tree based on the WGS of MPXV isolates from this study (extracted DNA samples have an “e” at the end of MPXVxx, whereas clinical samples have a “c” at the end), clustered with all published Swiss isolates (ON595760, ON622720, OP422245, OQ411312, ON792321, ON792322, OP783902), and references (OP715820, OP626136, OP415246, OP626135, OP523987, OQ451896, OP019276, OP484684, OR449644, OP523979) for each lineage and rooted using the A.1.1 isolate ON676708. The scale bar represents the number of substitutions per nucleotide. The colored point indicates the lineage to which the sequences belong to.

To determine the genetic relationship of the different MPXV isolates, we generated a maximum likelihood tree using the previously generated 32 consensus sequences (Fig. 6). This showed that the samples are distributed to the lineages B.1, B.1.1, B.1.4, B.1.5, and B.1.8. Combined with the samples in this study being collected over a period of 14 weeks, this indicates that there were likely multiple introductions of MPXV in the geographical area similar as to what was reported in other countries^9,12,13^. However, more importantly, sample sequences originating from different input matrices clustered all together, demonstrating that both matrices (i.e. extracted and clinical specimen) can be used as input. Combined, these results demonstrate that our established isothermal Phi29-based WGS method, with the alkaline lysis/denaturing step, can be directly applied to clinical material from patients diagnosed with mpox for rapid genomic surveillance of MPXV.

## Discussion

In this study, we present a novel whole genome sequencing method for MPXV using Phi29 DNA polymerase MDA-based amplification. We show that with only six octamers, each with multiple binding sites, combined with an alkaline denaturing step, we can resolve the whole genome sequence of MPXV from extracted nucleic acids from MPXV-positive lesions for phylogenetic analysis. Furthermore, using an alkaline lysis step, we demonstrate that this method can also be applied directly to clinical material from mpox lesions, which concordantly improves the sequencing coverage of the genomic ends of the MPXV genome. This novel isothermal whole genome sequencing approach allows the integration of whole genome surveillance of MPXV in any conventional laboratory setting.

Currently, the tiled-PCR WGS approach for MPXV allows the recovery of the almost complete genome and, combined with the sequencing library protocols, enables multiplexing, thus reducing overall sequencing costs^11,12^. However, the intricate dependency of a large array of primer pairs makes these protocols susceptible to genetic variations. Furthermore, achieving balanced amplification across the different amplicons requires individual primer pair calibration. However, in our approach, we reduced this risk by using octamers that anneal at multiple positions distributed over the genome and because the long elongation processivity (≥ 70 Kb) of Phi29 DNA polymerase creates an overall evenly distributed sequence coverage, except for the inverted terminal regions (ITR)^23^. Furthermore, we verified in silico that a similar octamer distribution is present in MPXV clade I (AF380138, NC003310) and clade IIa (KJ642616, AY753185) sequences. This indicates that the method described in this study can potentially be used for the genomic surveillance of different MPXV clades.

Our study shows that we could retrieve whole genome sequence information from clinical samples with a Ct-value ≤ 31. This is comparable with other whole genome amplification protocols for MPXV^11,12^. However, despite successfully isolating viable progeny virus from a sample with a Ct-value of 29.75, we had poor sequencing coverage for sample MPXV17 (Fig. 3, Supplementary Table S2). The reason for the poor sequencing coverage for this sample could be due to the relatively low viral load, sample degradation, pre-analytical specimen preparation procedure, or a combination thereof. Nevertheless, 88% of the genome could be recovered at a minimal sequencing depth of 1 nucleotide per position. This suggests that for some samples, an overall higher sequencing depth might be required, or virus isolation might be used as an intermediate step for WGS to increase the amount of starting material.

We demonstrate that clinical specimens from pustules and ulcers of mpox lesions can be used as direct input for our method. However, MPXV DNA has also been detected in blood, saliva, semen, urine, anorectal swabs, and fecal samples, and isolated from semen samples from mpox patients^24–28^. Hereby, it has also been shown that saliva can have similar viral loads as skin lesions, whereas the viral load in anorectal swabs peaks 4 days after the onset of symptoms and can even be detected up to 14 days^29^. However, whether these sample matrices can be used directly or indirectly after nucleic acid purification for WGS sequencing with the method described here still remains to be established. Similarly, it still remains to be determined whether this protocol can be used for genomic surveillance from samples of animal origin to identify potential animal reservoirs of MPXV.

In conclusion, we established a novel isothermal Phi29-based WGS methodology for MPXV that can potentially be applied to other sample matrices or viral families for genomic surveillance in virtually any conventional laboratory setting.

## Material & methods

### Clinical samples

In this study, we used 21 independent specimens that were sent for MPXV diagnostics to the clinical diagnostic laboratory of the Institute for Infectious Diseases, Bern, Switzerland. Monkeypox virus isolation from the specimens was conducted in a designated biosafety level 3 laboratory in line with national regulations (ECOGEN A220128). The Cantonal Ethics Commission Bern, Switzerland, approved the isolation and sequencing of Monkeypox virus isolates in clinical samples stored in the IFIK biobank (BASEC-Nr: Req-2023-00340).

## Cell culture

African green monkey kidney cells (Vero, American Type Culture Collection CCL-81, Manassas, VA, USA) were cultured in Dulbecco’s minimum essential medium containing Glutamax (DMEM + Glutamax; Gibco, Gaithersburg, MD, USA) and supplemented with 5% heat-inactivated Fetal Bovine Serum (FBS, PAN Biotech, Aidenbach, Germany), 100 µg/mL streptomycin, and 100 IU/mL penicillin (Gibco), 1% minimum essential medium non-essential amino acids (MEM NEAA (100×), Gibco), and 15 mM of N-2-hydroxyethylpiperazine-N-2-ethane sulfonic acid (HEPES, Gibco). The cell line was cultured at 37 °C in a humidified incubator with 5% CO_2_.

## MPXV isolation

One day prior to inoculation, Vero cells were seeded at a density of 1.66 × 10^5^ per well in a 12-well plate. Before infection, cells were washed twice with PBS (pH 7.4) and supplemented with infection media (DMEM with 2% FBS, 15 mM HEPES, and 100 µg/mL Primocin (InvivoGen, San Diego, CA, USA). Virus isolation was performed in a BSL-3 laboratory by centrifuging a total of 50 µL of clinical sample (1500 x rcf for 10 min), inoculating it to each well, and incubated at 37 °C in a humidified 5% CO_2_-incubator. After 72 h, cells were fixed using 4% buffered-formalin (Formafix Switzerland AG, Hittnau, Switzerland).

After fixation, samples were washed thrice with PBS and permeabilized overnight at 4 °C in CB buffer (PBS, pH 7.4, supplemented with 50 mM NH_4_Cl, 0.1% Saponin, and 2% BSA (IgG-free, Protease-free, Jackson ImmunoResearch, Westgrove, PA, USA)). Monkeypox virus-infected cell cultures were incubated with a primary antibody targeting the A27L protein (Vaccinia Virus (Lister Strain) Rabbit Polyclonal Antibody, OriGene, Rockville, MD, USA, 1:1000 in CB-buffer, also recognizing MPXV A27L protein) for 2 h at room temperature, followed by secondary antibody donkey anti-rabbit Alexa Fluor 488 (Jackson ImmunoResearch, 1:400 in CB-buffer) for 1 h at RT. The cell nuclei were counterstained using 4′,6-diamidino-2-phenylindole (DAPI, Thermo Fisher Scientific) for 5 min. The stained infected cells were visualized using a Cytation 5 Cell Imaging Multimode Reader (Agilent BioTek, Sursee, Switzerland) equipped with 2× (numerical aperture (NA): 0.05) air objective. The brightness of all the images was adjusted to the corresponding control using Fiji (v1.54 h), and the figures were generated and exported using the FigureJ plugin (v1.36)^30,31^.

### qPCR

To determine the best amplification settings for our protocol, we quantified the amplified DNA by qPCR. Briefly, the amplified samples were diluted 1:100 in nuclease-free water, and 2 µL was used as input for the qPCR reaction. The 10 µL reaction mixture was prepared using the Luna Universal Probe qPCR Master Mix (New England BioLabs (NEB), Ipswich, MA, USA) according to the manufacturer’s instructions. Previously published pan-Orthopoxvirus primers targeting the J7R gene were used, with one modification in the 13th nucleotide of the forward primer to address the mismatch to MPXV genomes (OPHA-F89_mod2: 5’-GAT GAT GCA ACT *M*TA TCA TGT A-3’)^32^. The analysis was done on a QuantStudio 7 Flex Real-Time PCR System (Thermo Fisher Scientific, Waltham, MA, USA) using an initial denaturation at 95 °C for 1 min, followed by 45 cycles of denaturation (15 s, 95 °C) and of annealing and elongation (30 s, 60 °C) followed by measuring the fluorescence intensity.

## DNA extraction

High-molecular weight (HMW) DNA was extracted from 200 µL of clinical sample using the ELITe InGenius SP 200 kit (ELITech Group, Puteaux, F), on an automated liquid-handling system (Biomek NGeniuS Next Generation Library Prep System, Beckman Coulter), and extracted nucleic acids were finally eluted in 100 µL according to the manufacturer’s protocol. The extracted DNA was then placed at -20 °C for short-term storage.

## Clinical sample and DNA pre-treatment

To denature the DNA prior to amplification, we incubated the extracted DNA or clinical specimen in an alkaline lysis buffer as previously described (0.4 M KOH, 10 mM EDTA, and 100 mM DTT, pH 14)^33^. Briefly, alkaline lysis buffer was added at a sample-to-buffer ratio of 0.85 and incubated for 5 min at 4 °C, after which the reaction was neutralized by adding an equal volume of 1 M Tris-HCl pH 4 (Promega). The pre-treated DNA was used as input for a multiple displacement amplification (MDA) using phi29 DNA Polymerase. A total of 5.64 µL of pre-treated DNA mixture were added to 15 µL of a reaction composed of 1x EquiPhi29 reaction buffer (Thermo Fisher Scientific), 0.6 U/µL EquiPhi29 polymerase, 1 mM dNTPs (Promega, Madison, WI, USA), 1 mM DTT, and 3.5 µM of pooled swga primers. The samples were incubated at either 42–45 °C for 1, 2, 3, or 4 h followed by enzyme inactivation (10 min, 65 °C).

### Primer design and isothermal amplification

The primers were designed using the command-line tool swga2.0 using MPXV (ON792320.1) as a reference and the human chromosomal and mitochondrial DNA (Homo sapiens GRCh38.p14 and Homo sapiens mitochondrion NC_012920.1) as the off-target template^34^. This resulted in multiple pools of approximately 7 octamers scored for binding frequency, mean distance between binding sites, coverage, and primer binding distribution, for both the on-target and off-target genome. Subsequently, we selected only octamers that in-silico did not align to the human mitochondrial DNA reference sequence (GRCh38:MT:1:16569:1 REF). This resulted in 6 octamers being selected (MPXV_01–06, Supplementary Table S1). The octamers were ordered at Microsynth (Microsynth AG, Balgach, Switzerland) with phosphorothioate bonds at the two 5’ terminal nucleotides, preventing exonuclease degradation. Each octamer binds multiple times across the MPXV’s genome in both the forward and reverse direction (ON792320.1, Supplementary Table S1); however, the first and last 600 bp of the viral genome were not included, and therefore, we custom-designed an octamer targeting both viral genomic ends (MPXV_ter2, Supplementary Table S1).

### Whole genome sequence analysis

Following isothermal amplification, MDA product was quantified using the Qubit 1X dsDNA Broad Range Assay Kit (Thermo Fisher Scientific) with a Qubit 4 fluorometer (Thermo Fisher Scientific). A total of 200 ng of the amplified product was used as input for nanopore sequencing library preparation using the rapid barcoding kit (SQK-RBK114.96, ONT, Oxford, UK) and sequenced with a MinION device (Mk1B, ONT) according to the manufacturer’s instructions, using either a Flongle (R10.4.1, FLO-FLG114) or a MinION flow cell (R10.4.1, FLO-MIN114, ONT) with super high-accuracy basecalling (Guppy 6.5.7, ONT) using the MinKNOW software (23.04.5, ONT).

Downstream sequence analysis was performed using wf-mpx pipeline from EPI2ME-labs (ONT), after which the figures were generated using R (v4.3.1). For the phylogenetic analysis all the generated consensus sequences were aligned using Nextclade Web with the Mpox virus (Lineage B.1) set as reference (https://clades.nextstrain.org)^35^. For phylogenetic tree generation, we manually removed the first 1500 bp from the 5’ end and the first 6422 bp from the 3’-end of the aligned sequences and then used them as input for IQ-TREE2 (version 2.2.6) using the built-in ModelFinder to select the best substitution model^36,37^. Finally, the maximum likelihood phylogenetic tree was generated using the HKY + I + F model with 1000 ultrafast bootstrap replicates^38^. Further image processing was done using the R package ggtree (v3.8.2)^39^.

## Supplementary Information

Below is the link to the electronic supplementary material.

Supplementary material 1 (PDF 1897.9 kb)

Supplementary material 2 (XLSX 10.6 kb)

Supplementary material 3 (XLSX 26.2 kb)

## Data Availability

Sequence data that support the findings of this study have been deposited in the European Nucleotide Archive with the primary accession code PRJEB73627 (https://www.ebi.ac.uk/ena/browser/view/PRJEB73627).
