# Author Correction: A novel isothermal whole genome sequencing approach for Monkeypox Virus

**DOI:** 10.1038/s41598-025-88455-w

**Published:** 2025-02-05

**Authors:** Matthias Licheri, Manon Flore Licheri, Lukas Probst, Cora Sägesser, Pascal Bittel, Franziska Suter-Riniker, Ronald Dijkman

**Affiliations:** 1 Institute for Infectious Diseases, University of Bern, Bern, Switzerland; 2 Graduate School for Cellular and Biomedical Sciences, University of Bern, Bern, Switzerland; 3 Multidisciplinary Center for Infectious Diseases, University of Bern, Bern, Switzerland; 4 European Virus Bioinformatics Center (EVBC), Jena, Germany

Correction to: *Scientific Reports* 10.1038/s41598-024-73613-3, published online 27 September 2024

The original version of this Article contained an error in the legend of Figure 1.

“Schematic overview of the two amplification workflows used in this study. Comparison of the two protocol workflows using either direct clinical material or extracted DNA as input. On the left the samples were extracted using an automated HMW DNA extraction system, followed by alkaline lysis buffer incubation and amplification, whereas samples on the right were directly lysed and then amplified. For both the workflows the amount of time needed for 24 samples is shown. Created with BioRender.com (https://app.biorender.com/, accessed on 5 February 2024).”

now reads:

“Schematic overview of the two amplification workflows used in this study. Comparison of the two protocol workflows using either direct clinical material or extracted DNA as input. On the left the samples were extracted using an automated HMW DNA extraction system, followed by alkaline lysis buffer incubation and amplification, whereas samples on the right were directly lysed and then amplified. For both the workflows the amount of time needed for 24 samples is shown. Created in BioRender. Licheri, M. (2024) BioRender.com/g83j532.”

The original Article has been corrected.

