# Supplementary material for: A novel isothermal whole genome sequencing approach for Monkeypox Virus
Source: Sci Rep. 2024 Sep 27;14:22333. doi: 10.1038/s41598-024-73613-3 (PMC11437064; doi:10.1038/s41598-024-73613-3)
Supplement: Supplementary file 1 — Supplementary material 1 (PDF 1897.9 kb) [file 41598_2024_73613_MOESM1_ESM.pdf]

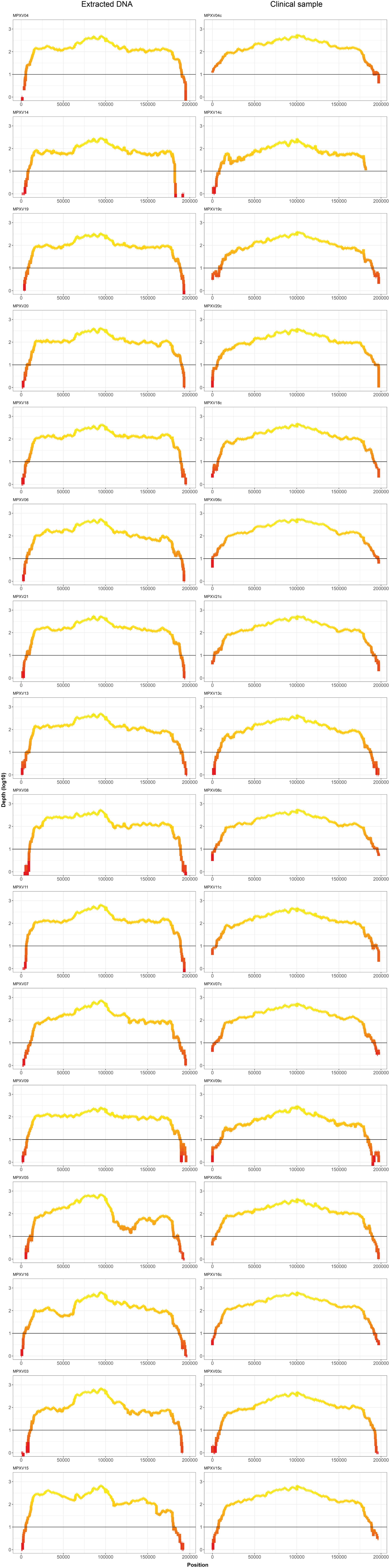
Extracted DNA
Clinical sample
MPXV04
MPXV04c
MPXV14
MPXV14c
MPXV19
MPXV19c
MPXV20
MPXV20c
MPXV18
MPXV18c
MPXV06
MPXV06c
MPXV21
MPXV21c
MPXV13
MPXV13c
MPXV08
MPXV08c
MPXV11
MPXV11c
MPXV07
MPXV07c
MPXV09
MPXV09c
MPXV05
MPXV05c
MPXV16
MPXV16c
MPXV03
MPXV03c
MPXV15
MPXV15c
0
1
2
3
0
50000
100000
150000
200000
Depth (log10)
Position

Figure S1: Comparison of the coverage achieved by samples amplified from extracted DNA and from clinical material. To compare the overall coverage of each sample, the achieved sequencing depth (log10 scale, y-axis) is plotted in function of each nucleotide position (x-axis) for the extracted DNA samples (left column) and the clinical material samples (right column). The black horizontal line corresponds to 10 times coverage.
